# Microfluidics and MEMS Technology for Membranes

**DOI:** 10.3390/membranes12060586

**Published:** 2022-05-31

**Authors:** Jasmina Casals-Terré

**Affiliations:** Department of Mechanical Engineering, Technical University of Catalunya, 08034 Barcelona, Spain; jasmina.casals@upc.edu

Nowadays manufacturing processes at nano and microscale provide reliable platform for the development of novel applications, specially in the membrane’s field. Separation systems based on nanoscale and microscale devices provide a superior control over the physico-chemical characteristics of the final product. Technology is capable for the first time to reproduce biological and physical properties and to use biomimetic approaches for separation or classification.

Microfluidic-based advanced membrane technology enables novel diagnostic tools and becomes the first step towards personalized medicine. Mature advances in this technology can potentially reduce the time-to-market of these necessary tools and exponentially reduce the turnaround time of diagnostic results. In addition, microfluidic-based separation strategies have been applied to different processes from wastewater treatment and agri-food to diagnostics. The potential of this technology is clear.

This Special Issue titled “Microfluidics and MEMS Technology for Membranes” in the journal *Membranes* aims to assess recent developments in microfluidics and MEMS that have a significant impact on membranes. Various topics are discussed, such as MEMS device manufacturing processes, microfluidic-based membranes, novel materials for nano separation, and novel applications in wearables. There are ten contributions, namely eight research articles and two reviews, in this Special Issue.

Hongfu Liang [1] et al. studied one of the key steps on electrohydrodynamic (EHD) printing, a cost-effective and environmentally friendly electrode manufacturing technology. EHD printing is a valued methodology to achieve ultrafine electrodes. In the paper, two methods of glass-surface modification were compared. Oxygen Plasma provided better results when combined with the appropriate annealing temperature. The silver electrodes decreasing to 20 um was a result achieved with the optimized methodology. This study paves the way for future separation methodologies that require electric fields such as electrophoretic-based membranes and others.

Honglong Ning [2] et al. had previously worked on the development of novel transparent flexible thin-film transistors (TFTs), key elements used in the field of transparent flexible flat-panel displays and in wearable sensors. Even though this technology is promising, it exhibited certain limitations, such as the elevated temperature required to deposit some layers. This requirement limited the number of substrate materials to be used and therefore the type of applications. In this work, they present a manufacturing process that fabricates such interesting devices at room temperature, exhibiting excellent performance and therefore allowing more flexibility in the kind of substrates to be used and opening the door to new applications.

Wenjun Zeng [3] et al. presents in his work a new driving waveform to reduce the oil film splitting in electrowetting displays. Electrowetting displays are analogous to cellulose in conventional paper. Today, electronic paper is a reality due to this new type of reflective device, which incorporates electrowetting displays. However, there are still issues to be solved, such as the limitations in the aperture ratio and the consumption. This work focuses on the study of new driving waveforms to achieve an optimized aperture ratio, while minimizing consumption.

Besides the improvement of manufacturing technologies, other works have focused more on the membrane development itself, either using novel materials or novel nanostructures. Muhammad Zahid [4,5] et al. introduced the use of nanomaterials such as sulfonated graphene oxide into cellulose acetate to enhance the hydrophilicity and anti-biofouling behavior to this new nanocomposite. These two improved characteristics are relevant for water treatment applications, especially when looking for high efficiency with minimal chemical use and cost effectiveness. On the other hand, Alvise Bagolini [6] et al. worked on the development of a neat membrane-in-membrane structure that used state-of-the-art manufacturing processes to etch nanoscale holes of free-standing membranes, while overcoming the previous limitations of pressure supported by this type of structure. The proposed structure can withstand up to 1 bar in pressure, having hole diameters from 300 to 600 nm. This characteristic enables a molecular flow regime that is of special interest for mass spectrometers and other analytical instruments.

There are other applications of membranes that do not require to focus at nanoscale, since the fluid dynamics at microscale can intrinsically separate particles from the suspending fluid. Rodríguez-Villarreal [7] et al. focused on the effect of temperature and flow rate to increase the efficiency of separation taking advantage of microfluidic hydrodynamics effects. The accuracy of the performance of microfluidic-based particle separators is essential to achieve an equivalent performance in point-of-care devices compared to the conventional laboratory-based analytics. Lin et al. [8] took advantage of a three-dimensional model developed with computer fluid-dynamics software (ANSYS-FLUENT) and with fluid-structure interaction coupling between fluid and solid domains to optimize a microfluidic membrane embedded in a microvalve. This work is an important tool to improve accuracy in microfluidic circuits; therefore, applications such as point-of-care devices benefit from the enhancement of the behavior of its components.

Currently, the aging of populations and the latest pandemic situation have overwhelmed most public-health systems. Governments are looking for new health strategies to overcome the current situation. E-Health and patient empowerment are two strategies that are being adopted. Rabost et al. [9], in the review, highlight the importance of sweat as an alternative fluid in following up treatments and measuring metabolites of interest. This is a driving strategy, but there is still a great deal of work to do in order to materialize this opportunity in the near future. New materials, processes, and studies of sweating processes through phantoms of skins are recent findings that are under investigation for in-depth understanding before upscaling for practical use. Versaci et al. [10] in their review examine membrane-based MEMS devices and the impact of the curvature of the membrane on its performance. Different analytical and modelling approaches are compared and evaluated according to their applicability to certain industrial devices.

In conclusion, the findings and critical discussions from these contributions highlight the importance of microfluidics and/or nanostructures for membrane development, manufacturing, and their use in novel applications that can be achieved in the near future. New materials, processes, and driving strategies demonstrate their various novel separation applications, and recent findings are under investigation for the in-depth characterization and upscaling for practical applications. This Special Issue introduces guidelines for the development of these new generation of membranes based on nano and micro features.
